# Liver Fatty Acid-binding Protein Is a More Reliable Biomarker for Liver Injury in Nonalcoholic Steatohepatitis than Other Etiologies of Hepatitis

**DOI:** 10.5152/tjg.2024.23444

**Published:** 2024-07-01

**Authors:** İlker Şen, Şükrü Dumlu

**Affiliations:** 1 Department of Gastroenterology, Şişli Hamidiye Etfal Education and Research Hospital, İstanbul, Türkiye; 2 Department of Gastroenterology, Gazi University Faculty of Medicine, Ankara, Türkiye

**Keywords:** Liver fatty acid-binding protein, viral hepatitis, nonalcoholic steatohepatitis, biomarker

## Abstract

**Background/Aims:**

Liver fatty acid-binding protein (LFABP) controls hepatocyte lipid metabolism and can be a biomarker in liver diseases. We compared the correlation of LFABP levels with liver histology in viral hepatitis and nonalcoholic fatty liver disease (NAFLD) and investigated the utility of serum LFABP as a biomarker for liver damage.

**Materials and Methods:**

We included 142 patients (60 chronic viral hepatitis B [CHB], 35 chronic viral hepatitis C [CHC], 47 NAFLD) and 40 healthy controls. LFABP levels were determined in all participants, and a liver biopsy was performed on patients. The nonalcoholic steatohepatitis (NASH) activity score (NAS), hepatosteatosis, liver inflammation, and fibrosis were evaluated for NAFLD patients. Ishak’s histological scores were used for viral hepatitis. The correlation between LFABP levels and histologic scores was assessed in each group.

**Results:**

Serum LFABP levels in CHB, CHC, NAFLD, and control groups were 2.2, 3.5, 7.6, and 2.1 ng/mL, respectively. LFABP levels were significantly higher in the NAFLD group compared to the control, CHC, and CHB groups. LFABP was significantly higher in the NASH group than in nonalcoholic steatohepatitis, 8 ng/mL and 5.4 ng/mL, respectively (*P =* .001). In the NAFLD group, LFABP levels showed a moderate positive correlation with NAS score (*r* = 0.58, *P* < .001), ballooning degeneration (*r* = 0.67, *P* < .001), and lobular inflammation (*r* = 0.62, *P* < .001). A logistic regression study showed that the level of LFABP was predictive of NASH independent of age, gender, homeostasis model of IR, body mass index, aspartate aminotransferase, and alanine aminotransferase (OR = 1.869, *P* = .01).

**Conclusion:**

LFABP specifically correlates with liver histology in NAFLD compared to viral hepatitis. Additionally, it can distinguish NASH from simple steatosis. LFABP may be a valuable biomarker for hepatocyte injury in NASH.

**Main Points**

- Liver fatty acid-binding protein (LFABP) plays an essential role in the transfer of free fatty acids, which is the pathogenic factor of in nonalcoholic fatty liver disease (NAFLD).
- This article indicates that serum LFABP levels were significantly higher in NAFLD patients, LFABP levels were also helpful to distinguish the nonalcoholic steatohepatitis from simple steatosis.
- Serum LFABP levels showed better correlation with histologic parameters in NAFLD patients compared to viral hepatitis B and C patients.

## Introduction

Nonalcoholic fatty liver disease (NAFLD) is a significant public health issue worldwide, with an increasing prevalence in parallel to the rising frequency of obesity and diabetes.^1^ Chronic viral hepatitis B (CHB) and chronic viral hepatitis C (CHC) are other significant causes of liver-related mortality, leading to severe complications.^2,3^

Despite the global burden of viral hepatitis and the increasing need to diagnose and grade NAFLD, there is yet no optimum noninvasive biomarker identified that could replace tissue sampling for evaluating liver damage.^4-6^

Fatty acid-binding proteins (FABPs) are highly expressed in all mammals and are mainly found in the cytoplasmic milieu.^7,8^ Liver fatty acid-binding protein (LFABP) is a soluble protein highly concentrated in hepatocytes and has a molecular weight of 15 kDa.^7^ In healthy human liver tissue, LFABP accounts for 7%-11% of cytosolic proteins in hepatocytes, making it a particular marker for the liver. In contrast, aminotransferases, commonly used as markers of hepatocyte damage, have a larger molecular weight of approximately 45 kDa. Although aminotransferases are produced in variable amounts in other tissues, LFABP is primarily expressed in the liver.^8^ When liver cells are damaged, serum levels of low molecular weight intracytoplasmic contents increase early and in excessive amounts, even with minimal cellular damage.^8,9^

Liver fatty acid-binding protein plays a crucial player in the absorption, transport, and metabolism of free fatty acids (FFAs), acting as a lipid chaperone that guides lipids and influences their biological functions.^10,11^ Liver fatty acid-binding protein regulates tissue-specific lipid signaling pathways, inflammatory responses, and metabolic control.^12^

In NAFLD, insulin resistance (IR) results in resistance in the antilipolytic effects of insulin, leading to an increase in FFA levels.^9,10^ In addition to the routinely used aminotransferases, studies offer LFABP as a more sensitive marker defining liver injury.^13,14^

Therefore, we hypothesized that serum LFABP levels might increase with hepatic damage, which is present in conditions such as NAFLD and viral hepatitis.

In this study, we aimed to investigate the possible correlation between serum LFABP levels and liver injury severity in patients with CHB, CHC, and NAFLD. We determined the correlation between LFABP levels and the histological and biochemical characteristics of these patients to evaluate whether LFABP could be a biomarker of hepatic damage.

## Materials and Methods

One hundred forty-two patients (60 CHB, 35 CHC, and 47 NAFLD) and 40 healthy control subjects were included in this study, with approval obtained from the Gazi University Ethics Committee for Non-Interventional Researches. (No: 193, date: May 9, 2012). Patients with other chronic liver diseases, biliary diseases, anemia, ischemic cardiac or cerebrovascular diseases, renal insufficiency, malignancy, a previous diagnosis of diabetes mellitus, alcohol consumption, drugs toxic to the liver, hormone replacement therapy, or herbal supplements were excluded from the study. Patients receiving drugs that could affect LFABP levels (lipid-lowering agents, angiotensin II receptor blockers, sitagliptin, or pioglitazone) were also excluded. The control group consisted of age- and sex-matched healthy volunteers with normal liver parenchyma on ultrasonography. Written informed consent was obtained from the patients who agreed to take part in the study.

Physical examination, anthropometric measurements, and laboratory tests were also performed. The weight and height of the participants were measured with a calibrated scale after patients had removed their shoes and any heavy clothing. Body mass index (BMI) was calculated as a person’s weight divided by height squared (kg/m^2^). Waist circumference was measured at the narrowest level between the costal margin and the iliac crest, while hip circumference was measured at the largest circumference around the buttocks. 

Standard laboratory parameters of all patients were routinely determined at the central laboratory of our center. Insulin resistance (IR) was estimated using the homeostasis model of IR (HOMA-IR) index, which was calculated by multiplying fasting plasma insulin (in microunits per milliliter) by fasting plasma glucose (in milligrams per deciliter) and dividing the product by 405. Subjects with a HOMA-IR score of 2.5 or higher were included in the IR subgroup. 

For LFABP analyses, all blood samples were collected from an antecubital vein between 8:00 and 9:00 AM after overnight fasting just before the liver biopsy procedure. Before analyzing LFABP, serum samples were centrifuged for 15 minutes at 1000 × *g* and then rapidly stored and frozen at –80°C until assayed. Following the manufacturer’s instructions, LFABP levels were measured using a commercially available enzyme-linked immunosorbent assay (ELISA) kit (USCN Life Science Inc., Houston, TX, USA).

Liver biopsy specimens were acquired using a 16-Gauge HepaFix needle (Braun, Melsungen, Germany) under ultrasonographic guidance. A single-blinded expert pathologist subsequently examined liver tissue samples and assessed the presence of steatosis, inflammation, and ballooning. Hematoxylin, Eosin, and Masson’s trichrome staining were used to evaluate the formalin-fixed and paraffin-embedded liver tissues.

The Ishak grading system was used to evaluate CHB and CHC cases. By combining the scores for each of the 4 necroinflammatory categories (portal inflammation: 0-4, focal necrosis: 0-4, confluent necrosis: 0-6, and interface hepatitis: 0-4), a histological activity grading score ranging from 0 to 18 was generated. Fibrosis was assessed separately on a scale of 0-6.^3^

Histopathological grading was based on the NAFLD scoring system established by the National Institute of Diabetes and Digestive and Kidney Diseases NASH Clinical Research Network.^15,16^ Steatosis was graded using the following scale: 0 (≤5%), 1 (≥5%-33%), 2 (≥33%-66%), and 3 (≥66%). Lobular inflammation was rated on a scale of 0-3, with 0 indicating no foci, 1 indicating ≤2 foci, 2 indicating 2-4 foci, and 3 indicating >4 foci. Ballooning was graded on a scale of 0-2, with 0 indicating none, 1 indicating a few ballooning cells, and 2 indicating many ballooning cells. According to Brunt’s criteria, steatosis (0-3), lobular inflammation (0-3), and ballooning (0-2) scores were computed, and these 3 scores were combined to determine the actual NAFLD activity score (NAS), which ranged from 0 to 8. Liver fibrosis was scored on a scale of 0-4, with 0 indicating no fibrosis, 1 indicating periportal or perisinusoidal fibrosis, 2 indicating perisinusoidal and portal/periportal fibrosis, 3 indicating bridging fibrosis, and 4 indicating cirrhosis.^15,16^ The mild fibrosis group included those with a fibrosis score of less than 2, while those with a fibrosis score of ≥2 were considered the significant fibrosis group. The NAFLD group was categorized as NASH or non-NASH based on the hepatopathologist’s assessment.

### Statistical Analysis

Statistical analysis was performed using the Statistical Package for the Social Sciences version 21.0 for Windows (IBM Corp., Armonk, NY, USA). The normal distribution of variables was assessed using both visual (histograms and probability plots) and analytical methods (Kolmogorov–Smirnov and Shapiro–Wilk tests). The Mann–Whitney *U*-test was used to compare variables that did not have a normal distribution. Student’s *t*-tests were used to compare the 2 study subgroups (NASH vs. non-NASH) for normally distributed continuous variables. The Kruskal–Wallis test was used to compare the HBV, HCV, NAFLD, and control groups. A value of *P* < .008, calculated using Bonferroni correction, was considered statistically significant. The correlations among the study variables were tested using Pearson’s and Spearman’s correlation coefficients according to the data distribution. The ability of LFABP to predict NASH was analyzed using receiver operating characteristic (ROC) curve analysis. When a significant cutoff value was observed, the sensitivity, specificity, positive predictive value (PPV), and negative predictive value (NPV) were determined. Multiple logistic regression analysis was performed to identify independent predictors of NASH. The possible factors identified using predictive markers, defined in previous studies and identified using univariate analysis, were further entered into logistic regression models. Model fit was assessed using Hosmer–Lemeshow goodness-of-fit statistics. Statistical significance was set at *P* < .05 (two-sided) and was considered statistically significant.

A modified frequency matching method was employed to match the control and patient groups. The exact percentages of male and female controls were accounted for during the matching process for sex. Furthermore, 3 distinct age ranges were established, and a comparable number of healthy individuals were enrolled and distributed among the 3 groups. The success of matching was subsequently verified through post-hoc analysis.

## Results

### Characteristics of Subjects

A total of 142 patients (60 CHB, 35 CHC, and 47 NAFLD) and 40 healthy controls were included in the study. The clinical and biochemical findings of the patients and controls are summarized in Table 1. The data revealed that all groups were well matched regarding age and sex. There were no significant differences in alkaline phosphatase (ALP), hemoglobin, platelet count, fasting plasma glucose (FPG), C-reactive protein (CRP), and triglyceride levels between the groups. However, total cholesterol, HOMA-IR, BMI, and waist and hip circumference values were significantly higher in the NAFLD group than in the control group. Only the hip circumference measurements revealed significant differences between the CHC and control groups. The other significant findings are presented in Table 1.

### LFABP Levels in NAFLD Group

**Figure 1.**
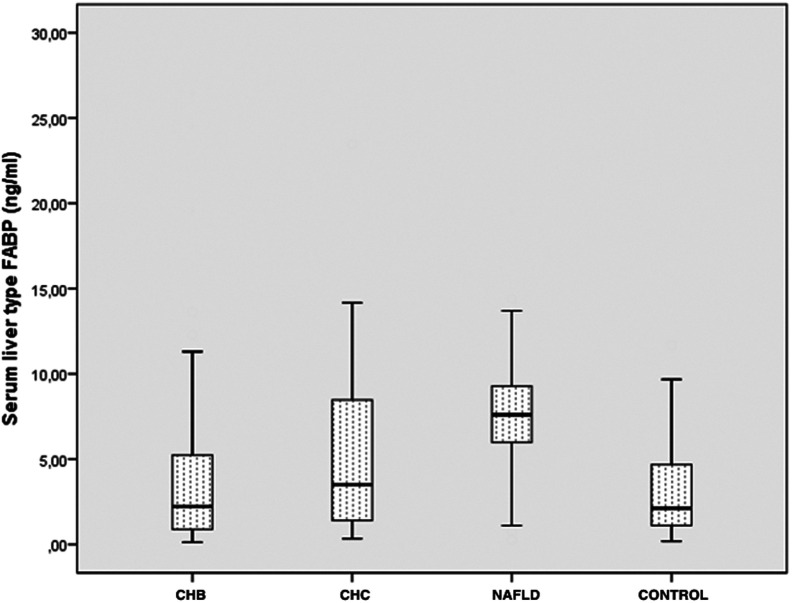
LFABP levels in CHB, CHC, NAFLD, and control groups. The graph shows interquartile range (box), median (thick line), range (thin lines), and outliers (circles) of plasma LFABP levels. The length of the box represents the interquartile range within which 50% of the values were located. CHB, chronic hepatitis B; CHC, chronic hepatitis C; NAFLD, nonalcoholic fatty liver disease; LFABP, liver aatty acid-binding protein.

**Figure 2.**
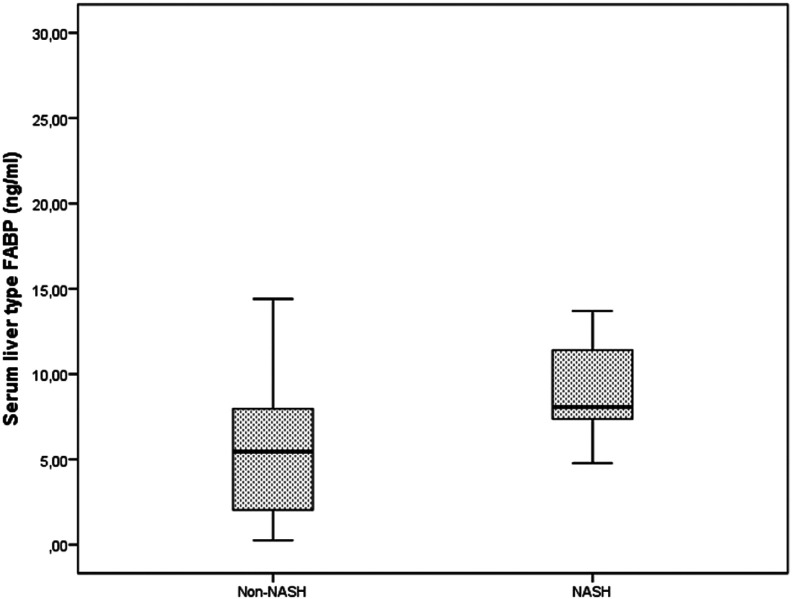
LFABP levels in NASH and non-NASH group. L-FABP serum levels increased significantly in NASH group (*P* = .001). FABP, fatty acid binding protein; NASH, nonalcoholic steatohepatitis.

The NAFLD group had significantly higher LFABP levels [7.6 ng/mL (5.7-9.4)] than the CHB and control groups (Figures 1 and 2). In the NAFLD group, LFABP levels showed a moderate positive correlation with the NAS score (*r* = 0.58, *P* < .001), ballooning degeneration (*r* = 0.67, *P* < .001), and lobular inflammation (*r* = 0.62, *P* < .001) (Table 2), a weak positive correlation with fibrosis, and no correlation with steatosis. In addition to histological parameters, HOMA-IR levels also showed a weak positive correlation with LFABP levels (*r* = 0.31, *P* = .03).

Liver fatty acid-binding protein levels in the NASH group (mean: 8, range: 7.3-11.5 ng/mL) were significantly higher than those in the non-NASH group (mean: 5.4, range: 1.4-8.5 ng/mL) (*P* = .001) (Figure 2).

### Correlation of LFABP Levels Between Laboratory Parameters and Anthropometric Measurements in NAFLD Group

No significant differences were observed in age, sex, BMI, hip circumference, HOMA-IR, FPG, total cholesterol, LDL, HDL, triglyceride, AST, ALT, GGT, ALP, or CRP levels between NASH and non-NASH groups (Table 3). Only waist circumference was significantly higher in the NASH group than in the non-NASH group (107.6 ± 11.1 vs. 100 ± 9.1, *P* = .02, respectively) (Table 4).

### Receiver Operating Characteristic Curve Analysis Results

**Figure 3.**
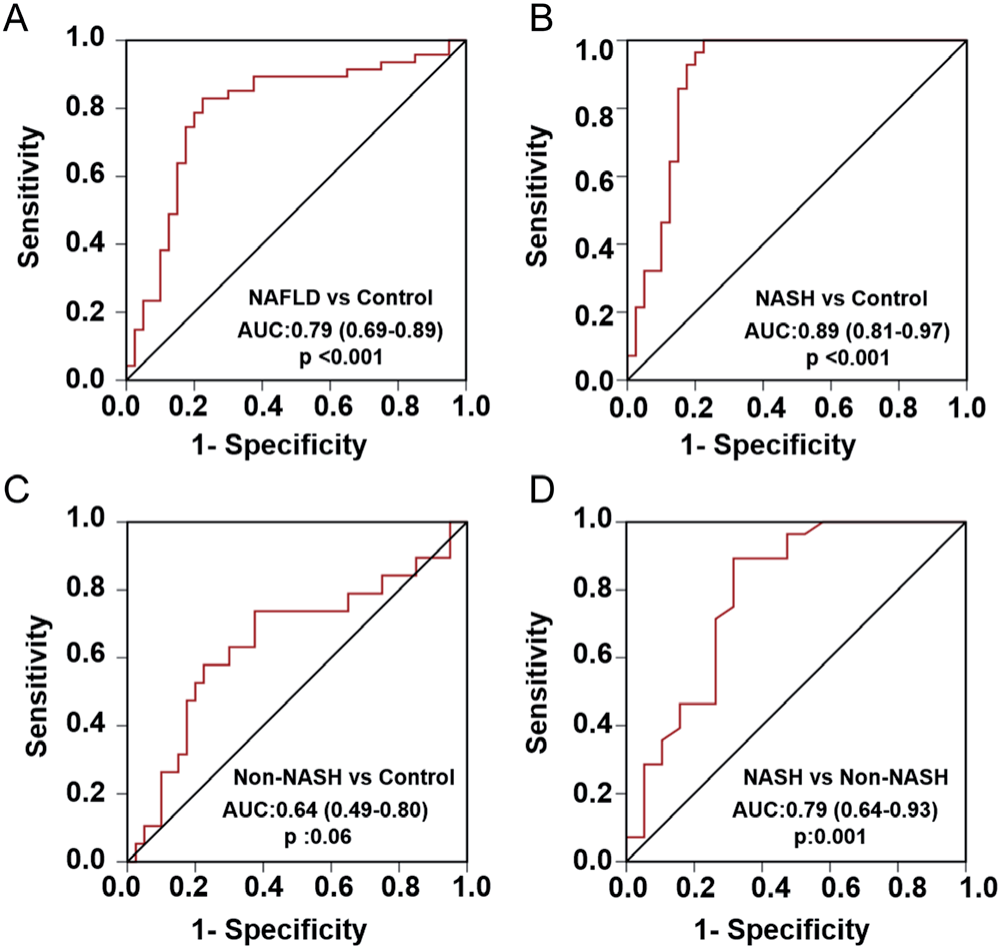
Receiver operating characteristic curves to differentiate (A) NAFLD vs. control, (B) NASH vs. control, (C) non-NASH vs. control, and (D) NASH vs. non-NASH groups according to the plasma L-FABP level (AUCs = 0.795, 0.895, 0.649, and 0 .790, respectively). LFABP, liver fatty-acid binding protein; NASH, nonalcoholic steatohepatitis; NAFLD, nonalcoholic fatty liver disease; AUC, area under the curve.

To identify the discriminatory significance of LFABP levels in patients with NAFLD, NASH, and non-NASH vs. controls, as well as in NASH patients vs. non-NASH patients, ROC curves were generated (Figure 3). Receiver operating characteristics analysis was conducted between the NAFLD versus control, NASH versus control, non-NASH vs. control, and NASH vs. non-NASH groups based on plasma LFABP levels. The AUCs were 0.795, 0.895, 0.649, and 0.790, respectively. The optimal cutoff value for distinguishing NASH from non-NASH was determined to be 6.395 ng/mL through ROC analysis, with a sensitivity of 89.3%, specificity of 68.4%, PPV of 80.65%, and NPV of 81.25%.

### Logistic Regression Analysis Results for Determining the Independent Predictors of NASH Within the NAFLD Group

The logistic regression analysis results showed that LFABP levels were predictive of NASH, independent of factors such as age, sex, HOMA-IR, BMI, AST, and ALT, with an OR of 1.869 and *P* value of .01 (Table 5).

### LFABP Levels in Viral Hepatitis Groups

In terms of LFABP levels, the CHB and control groups had similar levels [2.2 ng/mL (0.8-5.5) and 2.1 ng/mL (1-5.5), respectively]. The LFABP levels in the CHC group [3.5 ng/mL (1.3-9.1)] were moderately elevated compared to the control group, but the difference was not statistically significant. The results are presented in Table 1 and Figure 1.

In the CHB group, LFABP levels demonstrated a weak positive correlation with necroinflammatory activity (*r* = 0.36, *P* = .004), confluent necrosis (*r* = 0.35, *P* < .001), and fibrosis (*r* = 0.48, *P* < .001) (Table 6). Furthermore, in the CHC group, the levels of LFABP demonstrated a weak positive correlation with portal inflammation (*r* = 0.35, *P* = .04) and a moderate positive correlation with focal necrosis (FN) (*r* = 0.52, *P* = .001), necroinflammatory activity (*r* = 0.52, *P* = .001), and fibrosis (*r* = 0.65, *P* < .001). However, LFABP levels did not correlate with the viral load in either group.

Histopathologic findings of the CHB and CHC groups were as follows: In patients with CHB, 48 (80%) patients had a histological activity index (HAI) between 0 and 8, and 12 (20%) had a HAI between 9 and 18. The fibrosis scores were between 0 and 3 in 50 (83.3%) patients and 4-6 in 10 (16.6%) patients. In the CHC group, the HAI was between 0 and 8 in 23 patients (65.7%) and between 9 and 18 in 12 patients (34.3%). The fibrosis scores were between 0 and 3 in 30 (85.7%) patients and 4-6 in 5 (14.3%) patients in the CHC group.

## Discussion

Currently, there is a lack of an ideal, noninvasive biomarker for assessing liver tissue injury in the context of chronic viral hepatitis and NAFLD.^15-17^ Such biomarkers are necessary to accurately determine the severity of damage, predict treatment response, and understand their natural progression. Although the incidence of viral hepatitis has decreased, the prevalence of NAFLD is increasing globally. In the Cappadocia cohort study in Türkiye, hepatic steatosis was observed in 65.1% of men and 57% of women.^18^ Despite the growing number of studies on this topic, the primary challenge faced by these investigations is the inadequacy of biomarkers in detecting mild to moderate damage.^17,19^

This study aimed to establish a relationship between LFABP and histological and laboratory results in patients with CHC, CHB, and NAFLD. Moreover, we assessed the specificity of LFABP for different etiologies. This is the first study to reveal and compare the power of LFABP to determine the severity of liver damage in various liver diseases.

The increasing number of studies examining FABP family as a marker of damage for cholestatic liver disease, malignancies, diabetes, obesity, atherosclerotic conditions, and other specific tissues is noteworthy.^4,9,10,14,15,20^

Liver fatty acid-binding protein is a tissue-specific, low molecular weight protein that plays a crucial role in regulating intracytoplasmic lipid signaling pathways, as well as inflammatory and metabolic processes.^8^ It has been shown that the levels of LFABP are directly correlated with hepatic regeneration activity and cytoprotectant capacity.^7,9^ Pelsers et al^14^ suggested that LFABP might serve as a potential biomarker for detecting hepatocyte injury during the post-transplantation period. A study van den Broek et al^13^ demonstrated an increase in the levels of LFABP following the administration of the Pringle maneuver during liver surgery, while aminotransferase levels remained unchanged. Such results suggest that LFABP is a more sensitive marker to determine hepatocyte injury.

Several studies have evaluated hepatosteatosis in viral hepatitis.^21,22^ The distinct features of our study were the incorporation of 3 major causes of chronic liver disease (CHB, CHC, and NAFLD) and its goal of assessing the efficacy of a biomarker in biopsy-verified patients. Our findings indicated that serum LFABP levels were elevated in the NAFLD group compared to those in the control, CHC, and CHB groups, and this difference was statistically significant. Additionally, our results suggest that LFABP may help distinguish NASH from non-NASH in the NAFLD group.

Few studies have explored the levels of LFABP in chronic liver disease.^14,15^ However, to our knowledge, this is the first study to enroll patients with CHB and compare LFABP levels in CHB, CHC, and NAFLD patients with the findings of histological examination. Liver fatty acid-binding protein levels in the CHB group showed a mild-to-moderate correlation with necroinflammatory activity and fibrosis. However, there was no significant difference between the LFABP levels of the control and CHB group (2.1 and 2.2 ng/mL, respectively; *P* = .8).

Our study did not reveal significantly elevated levels of LFABP in the CHC group compared to controls. This result may be related to the high percentage of patients with mild-to-moderate histological findings in both the CHB and CHC groups in our study. However, correlation analysis between histologic findings and LFABP showed a stronger association in the CHC group than in the CHB group (necroinflammatory activity, *r* = 0.519, *P* = .001; fibrosis, *r* = 0.657, *P* < .001), in line with the study mentioned above.

Nonalcoholic fatty liver disease has become an attractive subject for recent studies because of its increasing incidence and high morbidity. The distinction of NASH is substantial in NAFLD, preferentially non-invasively, because of the potential progression of NASH to cirrhosis and hepatocellular carcinoma. In 22 biopsy-proven NAFLD patients, Higuchi et al^20^ found that hepatic expression of LFABP mRNA was upregulated in the NAFLD group compared to controls.

Only a single study has explored LFABP levels in patients with biopsy-confirmed NAFLD, and it reported weak positive correlations with histological parameters.^23^ Another study on NAFLD patients diagnosed based on elevated transaminases and steatosis detected through ultrasonography found higher LFABP levels in NAFLD patients compared to healthy subjects.^24^

The present study also revealed a positive correlation between LFABP level and ballooning degeneration, lobular inflammation, and fibrosis in the NAFLD group. Finally, LFABP levels were significantly higher in the NASH group than in the non-NASH group.

Atshaves et al^10^ emphasized the role of LFABP in obesity, but in the literature, there is insufficient data on LFABP levels in lean vs. obese NAFLD patients, and in obese patients with or without NAFLD, studies are needed to provide more accurate insights into these topics.

Liver fatty acid-binding protein is a vital endogenous cytoprotectant that protects hepatocytes from oxidative damage and mitigates ischemia–reperfusion and other hepatic injuries.^8^

According to data from in vitro and gene-ablated mice studies, LFABP may also play a vital role in the liver’s utilization of long-chain fatty acids (LCFAs).^10^

Alterations in LFABP levels impact lipid metabolism and oxidative stress within hepatocytes.^7,10,12^ Several studies have demonstrated that elevated levels of FFAs can play a role as a pathogenic factor in various metabolic disorders.^24^

One of the critical events in the progression of NAFLD is lipotoxicity, which arises from an excessive influx of FFAs into hepatocytes.^25,26^ Fatty acid-binding proteins transport FFAs to cell compartments and play a crucial role in cellular functions.^26,27^ These data illuminate the role of FABPs in hepatosteatosis and provide a rational basis for our findings.

The current scientific literature includes research on the utility of FABP family members, including LFABP, as biomarkers in various diseases and studies exploring the therapeutic potential of FABP inhibitors.^28^ These studies will provide additional support and verification of the outcomes of previous trials that sought to establish FABPs as reliable tissue damage markers.

The limitations of our study include the small sample size and potential bias in extrapolating the data to real-life patients owing to the exclusion criteria. Additionally, because our sample consisted of individuals of Turkish nationality, generalizing the results to other ethnic groups may not be appropriate. Because cirrhosis was an exclusion criterion, the same limitations were valid for patients with cirrhosis.

Considering these facts and the results of our study, we believe that serum LFABP levels increase with various liver injuries. This elevation is not solely the result of simple seepage through the damaged membrane but is also related to increased intracytoplasmic levels of LFBAP as a response to inflammation. The extent of LAFBP elevation is influenced by the etiology of liver injury.

In conclusion, our study indicated that LFABP levels were significantly higher in individuals with NAFLD than in the CHB, CHC, and control groups. Moreover, LFABP levels were positively correlated with histological scores in NAFLD, suggesting its potential use as a valuable marker for distinguishing between NASH and non-NASH cases. Although our findings indicate that LFABP may serve as a noninvasive biomarker for NASH, further studies are needed to establish LFABP as a reliable diagnostic marker for NAFLD and to determine its efficacy in differentiating between NASH and non-NASH.

## Figures and Tables

**Table 1.** Main Clinical and Biochemical Characteristics of the CHB, CHC, NAFLD, and Control Groups

	CHB (n = 60)	CHC (n = 35)	NAFLD (n = 47)	Control (n = 40)	*P*
Age (years)	44.8 ± 12.3	48.2 ± 8.3	46.1 ± 9.5	47.4 ± 15	.5
Male/female	25/35	14/21	18/29	19/21	.08
BMI, kg/m^2^	22.8 ± 2.2^¶^	24.6 ± 3.3	31.6 ± 4.5^#^^Δ^	22.5 ± 2.1	<.001
Waist circumference (WC), cm	76.3 ± 12.8	83.9 ± 20^#^	103.5 ± 10.4^¶Δ^	73.7 ± 12	<.001
Hip circumference (HC), cm	85.8 ± 12.7^†^	97.1 ± 16.1^#§§^	107.9 ± 7.9^¶Δ^	86.4 ± 13.6	<.001
Fasting plasma glucose (FPG), mg/dL	87 (78.5-96)^¶^	94 (80-98)	95 (88-112)	87.5 (78-96)^Δ^	.001
HOMA-IR	1.6 ± 0.6^¶^^††^	2.8 ± 2.3^§§^	4.7 ± 2.8^##Δ^	1.57 ± 0.6	<.001
AST, U/L	41 (38- 55)*	40 (33-58)^##§§^	49 (40-62)^Δ^	22 (18-24)	<.001
ALT, U/L	48.5 (40.2-58.2)^¶^*	43 (37-61)^#§§^	66 (48-91)^Δ^	18 (13.5-25)	<.001
GGT, U/L	32 (18.2-52.7)^¶^*	29 (20-46)	48 (39-81)^Δ^	20.5 (13.2-31)	<.001
ALP, U/L	80 (68.7-102.5)	90 (68-121)	76 (69-111)	80 (69.5-91.5)	.7
HGB g/dL	14.5 (13.1-16)^¶¶^	13.7 (13.3-14)	13.7 (13.3-14)	14 (13.8-14.2)	.02
WBC, mm^3^ (×1000)	5845 (4600-7300)	5245 (4490-7543)^##^	6539 (5430-7950)	6365 (5480-7700)	.01
PLT, mm^3^ (×1000)	196 ± 51^¶¶^	188.6 ± 70.7^##^	232.5 ± 63.5	225 ± 67.6	.02
Total cholesterol, mg/dL	174.7 ± 29**	189.5 ± 37.4^§^	204.5 ± 56.6^¶Δ^	159.3 ± 22.6	<.001
LDL, mg/dL	101.5 ± 28.4^¶^	115.2 ± 30.5^§^	128.8 ± 49	89.1 ± 27.3	<.001
HDL, mg/dL	48.3 ± 8.2	49.4 ± 12	45.7 ± 10.8^Δ^	51 ± 10.6	.1
Triglyceride, mg/dL	108.5 (93.7-145.7)^¶^	109 (76-154)	134 (92-187)	100 (53-123.7)^Δ^	.001
CRP, mg/L	1.6 (1.1-5.9)^¶^	2.5 (1.3-6.1)	4.2 (2.7-5.9)	1.3 (1.1-3.2)^Δ^	<.001
LFABP, ng/mL	2.2 (0.8-5.5)^¶^	3.5 (1.3-9.1)^ΔΔ^	7.6 (5.7-9.4)	2.1 (1-5.5)^Δ^	<.001

The column on the right represents *P* values obtained by the comparison of all 3 groups by Kruskal–Wallis test. A value of *P* < .008, calculated by Bonferroni correction, was considered statistically significant in the post hoc comparisons. Values are presented using mean ± standard deviations for normally distributed and medians and first and third quartiles (Q1-Q3) in the brackets for the non-normally distributed variables.

ALP, alkaline phosphatase; ALT, alanine aminotransferase; AST, aspartate aminotransferase; BMI, body mass index; CHB, Chronic Hepatitis B; CHC, Chronic Hepatitis C; CRP, C-reactive protein; GGT, gamma-glutamyl transferase; HGB, hemoglobin; HDL, high-density lipoprotein; HOMA-IR, Homeostatic Model Assessment-Insulin Resistance; LFABP, liver fatty acid-binding protein; LDL, low-density lipoprotein; MCV, mean corpuscular volume; MPV, mean platelet volume; NAFLD, nonalcoholic fatty liver disease; PLT, platelet; RDW, red cell distribution width; WBC, white blood cell.

*P < .01, CHB vs. control;

**P < .05, CHB vs. control;

^†^ *P* < .01, CHB vs. CHC;

^††^ *P* < .05, CHB vs. CHC;

^¶^ *P* < .01, CHB vs. NAFLD;

^¶¶^ *P* < .05, CHB vs. NAFLD;

^§^ *P* < .01, CHC vs. control;

^§§^ *P* < .05, CHC vs. Control;

^#^ *P* < .01, CHC vs. NAFLD;

^##^ *P* < .05, CHC vs. NAFLD;

^Δ^ *P* < .01, NAFLD vs. control;

^ΔΔ^ *P* < .05, NAFLD vs. control.

**Table 2.** Correlation Analysis of LFABP Levels with Respect to the Severity of Liver Histology in NAFLD

	*r*	*P*
Fibrosis	0.299	.04
NAS score	0.581	<.001
Ballooning degeneration	0.678	<.001
Steatosis	–0.072	.632
Lobular inflammation	0.620	<.001

LFABP, liver fatty acid-binding protein; NAFLD, nonalcoholic fatty liver disease, NAS, nonalcoholic fatty liver disease activity score.

**Table 3.** Main Clinical, Biochemical, and Pathological Characteristics of the NASH and Non-NASH Groups

	NASH (n = 28)	Non-NASH (n = 19)	*P*
Gender (M/F)	10/18	8/11	.6
Fibrosis	1 (0-3)	1 (0-1)	.2
NAS score	5 (5-6)	4 (2-5)	<.001
Ballooning	1 (1-2)	1	.01
Lobular inflammation	1.5 (1-2)	1 (0-1)	<.001
Steatosis	3 (2-3)	2 (1-3)	.1
FPG, mg/dL	92 (87-127)	98 (89-110)	.5
AST, U/L	49 (36-71)	50 (44-61)	.2
ALT, U/L	62.5 (41-91)	69 (54-91)	.3
ALP, U/L	92.5 (59-117)	76 (69-111)	.9
GGT, U/L	50 (31-81)	48 (39-70)	.6
HGB, g/dL	13.5 (13-13.7)	14 (13.6-14.6)	.08
PLT, mm^3^	217 (185-273)	230 (185-280)	.4
WBC, mm^3^	6.5 (5.7-7.9)	5.5 (5.4-7.9)	.2
BMI (kg/m^2^)	31.2 (±3.6)	31.9 (±5)	.6
WC, cm	107.6 (±11.1)	100 (±9.1)	.02
HC, cm	109.1 (±8.4)	107 (±7.5)	.3
Total cholesterol, mg/dL	209.5 (±43.9)	201.2 (±64.4)	.6
LDL, mg/dL	134.7 (±35.8)	124 (±56.6)	.5
HDL, mg/dL	45.9 (±11.5)	45.6 (±10.5)	.9
HOMA-IR	4.8 (±2)	4.6 (±3.2)	.8
Triglyceride, mg/dL	139 (95-200)	130 (92-172)	.6
CRP, mg/L	4.9 (3.2-6.8)	3.9 (2.7-5.9)	.4
FABP, ng/mL	8 (7.3-11.5)	5.4 (1.4-8.5)	.001

ALP, alkaline phosphatase; ALT, alanine aminotransferase; AST, aspartate aminotransferase; BMI, body mass index; CRP, C-reactive protein; FPG, fasting plasma glucose; GGT, gamma-glutamyl transferase; HOMA-IR, Homeostatic Model Assessment-Insulin Resistance; HC, hip circumference; HDL, high-density lipoprotein; HGB, hemoglobin; LDL, low-density lipoprotein; L-FABP, liver fatty acid-binding protein; MCV, mean corpuscular volume; MPV, mean platelet volume; NAFLD, nonalcoholic fatty liver disease; PLT, platelet; RDW, red cell distribution width; WC, waist circumference; WBC, white blood cell.

**Table 4.** Correlations in NAFLD Group with LFABP

	*r*	*P*
Age*	0.35	.014
Platelet*	0.298	.04
BMI	−0.135	.365
Waist circumference	−0.62	.6
Hip circumference	0.035	.8
Total cholesterol	0.05	.7
LDL	0.03	.8
HDL*	0.09	.5
HOMA-IR	0.314	.03
Fasting plasma glucose	0.18	.2
Triglyceride	0.25	.08
C-reactive protein	0.07	.5
AST	−0.17	.2
ALT	0.09	.5
ALP	0.16	.2
GGT	0.03	.8

ALT, alanine aminotransferase; AST, aspartate aminotransferase; ALP, alkaline phosphatase; BMI, body mass index; GGT, gamma-glutamyl transferase; HDL, high-density lipoprotein; HOMA-IR Homeostatic Model Assessment-Insulin Resistance; LFABP, liver fatty acid-binding protein; LDL, low-density lipoprotein; NAFLD, nonalcoholic fatty liver disease.

*Pearson correlation performed for parameters, Spearman correlation applied for all others.

**Table 5.** Logistic Regression Analysis to Determine the Independent Predictors of NASH Within the NAFLD Group

Factor	OR	95% CI for OR		*P* value
		Lower	Upper	
LFABP	1.869	1.287	2.712	.001
NASH	0.888	0.795	0.991	.034
BMI	1.256	1.004	1.570	.046
ALT	0.959	0.919	1.002	.062

ALT, alanine transaminase; BMI, body mass index; LFABP, liver fatty acid binding protein, NASH, nonalcoholic steatohepatitis.

**Table 6.** Correlation Analysis of LFABP Levels with Respect to the Severity of Liver Histology in CHB and CHC

	CHB		CHC	
	*r*	*P*	*r*	*P*
Necro-inflammatory activity (NIA)	0.364	.004	0.519	.001
Focal necrosis (FN)	0.126	.337	0.375	.027
Confluent necrosis (CN)	0.358	.005	0.466	.005
Portal inflammation (PI)	−0.304	.018	0349	.04
Interface activity (IA)	0.365	.004	0.292	.210
Fibrosis	0.485	<.001	0.657	<.001

CHB, chronic hepatitis B; CHC, chronic hepatitis C; LFABP, liver fatty acid-binding protein.
